# Design and Characterization of a New Quercus Suber-Based Pickering Emulsion for Topical Application

**DOI:** 10.3390/pharmaceutics11030131

**Published:** 2019-03-19

**Authors:** Catarina Carriço, Pedro Pinto, Angélica Graça, Lídia Maria Gonçalves, Helena Margarida Ribeiro, Joana Marto

**Affiliations:** 1 Research Institute for Medicine (iMed.ULisboa), Faculty of Pharmacy, Universidade de Lisboa, 1649-003 Lisbon, Portugal; catarinaalmeidacarrico@gmail.com (C.C.); geral@phdtrials.com (P.P.); angelicagraca@campus.ul.pt (A.G.); lgoncalves@ff.ulisboa.pt (L.M.G.); hribeiro@campus.ul.pt (H.M.R.); 2 PhD Trials, Rua das Murtas, nº1B, 1º, 1700-309 Lisboa, Portugal

**Keywords:** *Quercus suber* L., Quercus Suber Bark particles, Pickering emulsion, anti-oxidant activity, Quality by Design approach

## Abstract

Quercus Suber Bark from *Quercus suber* L. is a natural, renewable and biodegradable biomaterial with multifunctional proprieties. In this study, we used it as solid particles to stabilize a Pickering emulsion. The main goal was to produce an optimized topical formulation using biocompatible organic particles as stabilizers of the emulsion instead of the common surfactants, whilst benefiting from *Quercus suber* L. proprieties. In this work, a Quality by Design (QbD) approach was successfully applied to the production of this emulsion. A screening design was conducted, identifying the critical variables of the formula and process, affecting the critical quality attributes of the emulsion (droplet size distribution). The optimization of the production was made through the establishment of the design space. The stability was also investigated during 30 days, demonstrating that Quercus Suber Bark-stabilized emulsions are stable since the droplet size distribution lowers. In vitro studies were performed to assess antioxidant and antiaging efficacy, which revealed that the formulation had indeed antioxidant proprieties. A physicochemical characterization demonstrated that the formulation presents a shear-thinning fluid, ideal for topical administration. The in vivo compatibility study confirmed that the final formulation is not skin irritant, being safe for human use. A sensorial analysis was also performed, using a simple sensory questionnaire, revealing very positive results. Thus, the use of Quercus Suber Bark particles as a multifunctional solid ingredient contributed to achieve a stable, effective and innovative Pickering emulsion with a meaningful synergistic protection against oxidative stress.

## 1. Introduction

Emulsions are thermodynamically unstable, their study and development being some of the most difficult and complex subjects in the pharmaceutical and cosmetic fields. Emulsions emerge as a good solution for topical drug delivery. Its main interest is to encapsulate a hydrophilic or lipophilic active molecule inside the dispersed phase, ensuring its protection against environmental stress and degradation and allowing its controlled delivery [1,2,3].

Emulsions are most commonly stabilized by synthetic surfactants, some of which can even raise environmental issues and be intrinsically toxic [3,4].

Pickering emulsions, on the other hand, are surfactant-free liquid or semi-solid systems stabilized by solid particles. Consequently, these solid-stabilized emulsions constitute an interesting alternative strategy for encapsulating drugs in topical pharmaceutical formulations [5]. This type of emulsion was named after Spencer U. Pickering, whose paper first described the phenomenon in 1907. This stabilization of emulsion droplets is due to particle’s dual wettability: partial wetting of the surface of the solid particles by water and oil is the origin of the strong anchoring of these particles at the oil–water interface [6,7]. Wettability allows the spontaneous accumulation of particles at the mentioned interface, which is stabilized against coalescence by volume exclusion and steric hindrance [8].

In these emulsions, one of the liquids will wet the solid particles more than the other. The liquid with the poorest wetting properties is considered the disperse phase. The type of emulsion is determined by the contact angle (θ) between the particle and the interface, which quantifies the wettability of the particles at the oil–water interface. Contact angles lower than 90° give rise to o/w emulsions while contact angles higher than 90° favour w/o emulsions. However, if the particles have very high contact angles (too lipophilic) or very low ones (too hydrophilic), they will tend to be dispersed in either the oil or aqueous phases, respectively, leading to very unstable emulsions [1].

The effectiveness of the solid particles in acting as Pickering emulsion stabilizers depends on particle size, shape, concentration, wettability and interactions between particles. The particles should be substantially smaller than the targeted emulsion droplet size. In addition, small sized particles have the advantage of reducing the amount required to stabilize a given emulsion droplet interface [1,8,9]. Various types of solid particles can be used in Pickering emulsions including organic, such as polymer latex or starch, or inorganic, such as silica, titanium dioxide and clay particles. Nowadays, novel delivery systems such as nanoparticles and cyclodextrins (CD) are also being studied and developed [7,10,11].

The most common particle stabilized emulsion systems tested today are based on inorganic particles. There is a wide range of commercially available particle sizes (from a few nanometers to several microns), surface areas, and adjustable hydrophobicity (improved by changing the particle coating, the extent of chemical modifications, or the degree of substitution by functional groups). Different types of inorganic-based particles have been successfully used and recognized as efficient stabilizers for topical Pickering emulsions, such as silica and Titanium dioxide (TiO_2_), among others [9,10,12].

Presently, there is a strong market trend towards the formulation of green and natural products, creating a huge variety of pharmaceuticals and cosmetics. This motivates companies to develop particles based on material derived from plants or microorganisms, that is, renewable resources, meeting the demand for more eco-friendly products. The biocompatibility and the environment-friendly characteristic of organic particles constitute a great advance in healthcare and pharmaceutical products [13,14]. Starch and CDs are some of the new sources of particles used for stabilizing Pickering emulsions [5,6].

Surprisingly, another type of organic material that can be used as a Pickering emulsion stabilizer is Quercus Suber Bark (QSB), more commonly known as Cork. Cork is a natural material obtained from the outer bark of Quercus suber L. Cork is a lightweight material with other characteristic such as being impermeable to liquids, a good thermal insulator, resistant to microbial activity and presents a high friction coefficient [15]. It is a renewable and biodegradable raw bioresource concentrated mainly in the Mediterranean region [16]. It consists essentially of suberin, lignin and cellulose, containing also a small amount of extractives, fatty acids, terpenes, long chain aliphatic compounds and saccharides. The presence of several extractable phenolic acids was also identified. 

The interest on these natural phenolic compounds relies on the wide variety of relevant properties shown by this family, namely, their antioxidant, anti-inflammatory, radical scavenger and antimicrobial properties. Several studies prove that the extracts obtained from QSB have in fact anti-oxidant, anti-ageing, anti-inflammatory and anti-fungal properties all from a naturally occurring and sustainable material [15].

This heterogeneity of chemical composition and its extraordinary properties make QSB a material with a lot of potential and considerable importance in several industries [17,18]. Moreover, like other natural compounds, they do not show many of the adverse effects frequently shown by their synthetic counterparts. Altogether, this justifies the considerable growing interest on its use for pharmaceutical, nutraceutical and cosmetic applications [15,17,19,20].

The aim of this work was to develop and optimize an emulsion stabilized by QSB particles, meeting the demands of the consumers for green and natural products, using biocompatible organic particles in Pickering emulsions for high skin compatibility, whilst profiting from QSB proprieties such as antioxidant and antiaging capacity. The physicochemical stability, namely, droplet size distribution, of such emulsions was assessed. The in vitro efficacy was also studied, in particular the antioxidant activity, using HaCat cell cultures and antiaging capacity. In vivo safety and compatility properties of the final formulation, including the Human Repeat Insult Patch Test (HRIPT) were also evaluated.

## 2. Materials and Methods

### 2.1. Materials

Quercus Suber Bark (PureCork B 100) (QSB) was obtained from Amorim Cork Composites (Santa Maria da Feira, Portugal). The oils used were Liquid Paraffin obtained from António M. S. Cruz, Material de Laboratório, Lda. (Lisbon, Portugal), caprylic/capric acid triglyceride (Tegosoft® CT) (CT), phenoxyethyl caprylate (Tegosoft® XC) (XC), dimethicone (Abil® 350) and cetyl dimethicone (Abil® Wax 9840), the last four a kind gift from Evonik Industries AG (Essen, Germany). 1,2-Hexanediol (Dermosoft® Hexiol) were obtained from Dr. Straetmans (Hamburg, Germany). Sodium acrylates copolymer (and) Lecithin (Lecigel™) were obtained from Lucas Meyer Cosmetics (Champlan, France). Purified water was obtained by reverse osmosis and electrodeionization (Millipore, Elix 3) being afterwards filtered (pore 0.22 µm). 2′,7′-dichlorodihydrofluorescein diacetate (H2-DCFDA) was from Life Technologies (Carlsbad, CA, EUA), and spontaneously immortalized human keratinocyte cell lines HaCaT (CLS, Eppelheim, Germany) were used for antioxidant activity and cytotoxicity assays with cells.

### 2.2. Methods

#### 2.2.1. Quercus Suber Bark (QSB) Particle Size Distribution

Particle size distribution was determined using a Malvern Mastersizer 2000 (Malvern Instruments, Malvern, UK), coupled with a Hydro S accessory. The refractive index used was 1.52 (default). For a correct turbidity, corresponding to an obscuration between 10–20%, the sample was added to the chamber containing purified water. The readings were performed under mechanical stirring (1750 rpm) and ultrasounds (25%). Data was expressed in terms of relative distribution of volume of particles in the range of size classes, and given as diameter values corresponding to percentiles of 10, 50 and 90. The Span value is a useful statistical parameter to characterize the particle size distribution, and it was calculated using Equation [5]:

(1) $$\mathrm{Span}=\frac{d\left(90\right)-d\left(10\right)}{d\left(50\right)}$$

#### 2.2.2. Wettability Measurements of QSB Particles

The contact angles of the samples were measured by means of the Wilhelmy plate technique, using a Tensiometer K12 (Krüss GmbH, Hamburg, Germany). For applying this technique, a rectangle shape substrate coated with a double-side tape (20 × 20 mm) was used and the powder was coated onto its surface. It was ensured that the tape was uniformly coated with powder so that there was no tape exposed. The solid sample (QSB particles) was hung perpendicular to the liquid surface. Five different liquids were used: water, caprylic/capric acid triglyceride (CT), phenoxyethyl caprylate (XC), dimethicone and cetyl dimethicone. Each liquid was placed in a clean glass dish and raised by means of a motorized platform to contact with the powder plate. The platform was raised at the speed of 6 mm/min and the submersion distance was 2 mm. The volume of liquid used for the contact-angle measurements was ca. 50 cm^3^. The contact angle was calculated from the measured force by transposing the Wilhelmy equation using Krüss Laboratory Desktop Software (version 3.2, Krüss GmbH, Hamburg, Germany).

#### 2.2.3. Preparation and Characterization of Emulsions Stabilized by QSB Particles 

QSB particles were firstly dispersed in the caprylic/capric acid triglyceride. The oil and aqueous phases were then mixed using an UltraTurrax® homogenizer (IKA-Werke GmbH & Co. KG, Staufen, Germany) at 12,000 rpm during 5 min at room temperature (cold process). Using a light microscope (Olympus BX51, Shinjuku, Japan) with 10× objectives with normal light, we verified the existence and shape of the emulsions’ droplets.

#### 2.2.4. Droplet Size Analysis

The size distribution of the emulsions’ droplets was determined using the same method described in Section 2.2.1. In order to achieve an acceptable turbidity, each sample, corresponding to an obscuration between 10–20 %, was added to the sample chamber containing filtered purified water using a stirrer at 700 rpm during 30 s.

#### 2.2.5. Optimization Studies

##### Identification of Quality Target Product Profile (QTPP) and Critical Quality Attributes (CQAs) 

The first and most important element when using the QbD approach to assist formulation and process design is to pre-define the desired final QTPP (Table 1). The QTPP describes the product quality and forms the basis for defining the CQAs and critical process parameters (CPPs). The first step is to define the desired QTPP, which depends on scientific, regulatory and practical considerations and previous work. One of the important features of an emulsion is its droplet size distribution, namely, d(10), d(50), d(90) and span, because it influences other important characteristics such as rheology and stability. 

#### 2.2.6. Risk Analysis of CQAs

The first step in the risk assessment was to gather all the possible factors that could influence product quality. For this purpose, the identification of critical variables and the levels used in DoE were based on the literature and previous work. With the information collected, Ishikawa diagrams were constructed to identify the potential risks associated to emulsion stability as well as the process parameters, identifying the CQAs that have the greatest chance of generating product failure. The droplet size was defined and further delineated to identify potential risks and after the analysis; two variables were identified for optimization in the following studies.

#### 2.2.7. Design of Experiments (DoE)

The formula of the emulsions was optimized using a two-factor Central Composite Design (CCD). The independent variables for the process optimization had already been studied in previous work [5] and were not, for that reason, analysed here. For the formula optimization, the percentage of oil phase and the percentage of QSB particles were the independent variables analyzed.

To investigate the variables affecting the responses studied, we used a central composite design composed of five levels, coded as −α, −1, 0, 1, and +α, for the formula with CT as the oil phase. The value for alpha (1.147) was chosen in order to ensure design rotability.

This design required 11 experimental runs (Table 2) with three replicated centre points for more uniform estimate of the prediction variance over the entire design space. The data was analysed using the MODDE® Pro 11 software (Umetrics, Umeå, Sweden) and statistical analysis was considered significant when the estimated *p*-values were lower than an α error of 0.05.

Then, the response was studied, which means the droplet size distribution of all 11 experimental runs. The size distribution of the emulsions’ droplets was measured by light scattering using a Malvern Mastersizer 2000 (Malvern Instruments, Worcestershire, UK) coupled with a Hydro S accessory as previously detailed (Section 2.2.1.).

#### 2.2.8. Stability Assessment of Optimized Formulation

Based on the QbD approach, an optimized formulation stabilized by QSB particles (QSB-stabilized emulsion) was created. To assess the stability of this optimized formulation, 3 batches with the same conditions were prepared and their respective droplet size distribution measured at different times, for 1 month (0, 14 and 30 days).

#### 2.2.9. In Vitro Studies

##### Cytotoxicity Assay

The cytotoxicity was evaluated using general cell viability endpoint MTT reduction (3-(4,5-dimethyl-2-thiazolyl)-2,5-diphenyl-2*H*-tetrazolium bromide) assay. Cell viability was assessed after 24 h of incubation of a spontaneously immortalized human keratinocyte cell line HaCaT (CLS, Eppelheim, Germany) with different concentrations of QSB-stabilized emulsion (100 μg/mL to 1 μg/mL) and the procedure was performed according a previous published work [6].

##### Anti-Oxidant Activity

The intracellular production of reactive oxygen species (ROS) within cells was evaluated with a fluorimetric technique using 2,7’ dichlorodihydrofluorescein diacetate (H2-DCFDA). HaCaT sub-confluent cells grown in 96 well plates were incubated for 30 min with 20 μM of H2-DCFDA in the dark, at 37 °C and the procedure was performed according a previous published work [23]. Data from 12 replicates was reported as relative fluorescence units (RFU) percentage and expressed as a mean fluorescence ratio (fluorescence of exposed cells/fluorescence of unexposed control from the same experiment). The normal cell medium was used as negative control and ascorbic acid (AA) was used as positive control. These results were reported as the mean ± standard deviation.

##### Enzymatic Inhibition Assay

Fluorometric assays for the QSB-stabilized emulsion inhibition activity were carried out in 200 μL assay buffer (0.1 M HEPES pH 7.5 at 25 °C) containing 20 μL of 0.17 μM HNE (stock solution 1.7 μM in 0.05 M acetate buffer, pH 5.5), 155 μL of assay buffer and 5 μL of each concentration of tested inhibitors. After 30 min of incubation at 25 °C, the reaction was initiated by the addition of 20 μL of fluorogenic substrate to a final concentration of 200 μM (MeO-Suc-Ala-Ala-Pro-Val-AMC, Merck, Darmstadt, Germany). The Michaelis–Menten constant (*K*_m_) of this substrate of HNE was previously determined to be 185 μM (data not shown). For all assays, saturated substrate concentration was used, throughout, in order to obtain linear fluorescence curves. Controls were performed using enzyme alone, substrate alone, enzyme with DMSO and a positive control (Sivelestat sodium salt hydrate, Sigma Aldrich, St. Louis, MO, EUA) [23].

#### 2.2.10. Physicochemical Characterization

The pH measurements were performed with the pH-meter (S20 Seven easy pH, Mettler Toledo, Columbus, OH, EUA) and the data collected after 5 min at room temperature. Six replicates of the measurements were performed for the emulsion.

The apparent viscosity and rheological profile of QSB-FF were evaluated at room temperature, using a Brookfield Rotation Viscosimeter®, RV DV-II, SSA with a spindle 07 (Brookfield Engineering Laboratories, Middleboro, MA, USA ) immersed into the sample of the emulsion. The shear rate [1/s] versus shear stress [Pa] plots (flow curves) were obtained by submitting the samples to a shear rate sweep from 12.24 to 122/s up (ascent curve) and down (descent curve). This means that the angular velocities varied from 10 rpm to 100 rpm and each one was read after 30 s, and then reversed the velocity to the initial. 

#### 2.2.11. In Vivo Compatibility and Sensorial Studies

##### Human Repeat Insult Patch Test (HRIPT)

A safety evaluation study (Ref. 4971216.L, 01/2017) was performed on the QSB-stabilized emulsion, using the Marzully and Maibach [24] HRIPT protocol. Briefly, the product was applied on the back of 51 healthy volunteers that gave their prior informed written consent. For the induction period, a series of nine patches (Finn Chamber standard) were performed over a period of 3 weeks. Reactions after patching were scored according to International Contact Dermatitis Research Group. The results are expressed in score of skin irritation, calculated from the “marks” allocated to the visible signs: erythema, oedema, vesicle, papule (from 1 to 2 or 3) and bulla, scab (2 if presence) which takes into account the intensity of skin reactions. For each subject (and for each observation time), an individual daily irritation score was calculated: sum of all the marks obtained for the observed signs. A 2 week-rest period was maintained without application of the test emulsion. During the challenge period, new patches were prepared and fixed in the same manner as in the induction period, but also on the right side of the back (i.e., a virgin site).

The protocol was approved by the local Ethical Committee (MS/2017/4457/P22315) and respected the Helsinki Declaration and the AFSSAPS regulations on performed HRIPT studies on cosmetic products. The study was conducted under the supervision of a dermatologist who participated in the evaluation of irritation/allergic reactions to the emulsions.

##### Sensorial Analysis

Thirty-eight volunteers were questioned to scale characteristics of the QSB-stabilized emulsion. An inquiry was presented to them and each volunteer answered a few questions about the emulsion sensory attributes, such as fragrance, consistency and stickiness, skin feel during application (ease of application, spreadability and oiliness), skin feel after application (hydration, softness of the skin and freshness) and the willingness to buy the product. Responses were given in a scale from 1 to 4. Sensory parameters were evaluated by applying a small amount of QSB-stabilized emulsion in the back of their hands and rubbed it into the skin until it totality disappeared.

#### 2.2.12. Statistical Analysis

Results are expressed as mean values ± standard deviation (SD). Statistical evaluation of data was carried out using GraphPad Prism v. 5.02 (GraphPad Software, San Diego, CA, USA) by one-way ANOVA (Analysis of variance), by a Tukey’s comparison test in the in vitro studies, with significance set at *p*-values <0.05.

## 3. Results and Discussion

### 3.1. Formulation Studies

#### Quercus Suber Bark (QSB) Particle Size Distribution

**Figure 1:**
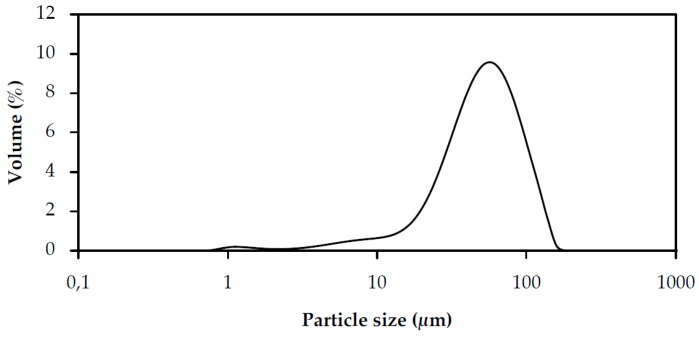
Quercus Suber Bark (QSB) particle size distribution (mean, *n* = 6).

It is important to work with particles with size distribution higher than 100 nm in order to avoid regulatory issues, including the need to perform genotoxicity studies and to prevent nano-ethical problems [25]. The particle size distribution of QSB particles can be defined as a Normal-Gaussian distribution and a mono-modal one, both facts indicating a more stable and uniform distribution [26]. The QSB particles showed a particle size ranging from 18.6 ± 0.0 (d10) to 91.4 ± 0.3 µm (d90), with a main peak at 47.4 ± 0.1 µm; furthermore, it is evident that most particles were smaller than the targeted emulsion droplet size (Figure 1). This supports the use of QSB particles as effective stabilizers of emulsions. In addition, small sized particles have the advantage of reducing the amount required to stabilize a given emulsion droplet interface and granting a smaller droplet size to that emulsion. 

### 3.2. Wettability Measurements of QBS Particles

According to Bancroft Rule, the emulsion type is often related to the preferential solubility of the emulsifying agent in one of the phases, which means the phase in which the stabilizing agent is more soluble will be the continuous phase, those preferentially soluble in water stabilize o/w systems and vice versa [27]. In this surfactant-free system stabilized by solid particles, one of the liquids will probably wet the solid more than the other liquid, with the more poorly wetting liquid becoming the disperse phase. The importance of the wettability of the particles at the oil–water interface is quantified by the contact angle, θ, the angle that the particle makes with it, which will determine the type of emulsion. If the contact angle, measured through the aqueous phase, is inferior to 90° the emulsion will be o/w and, by contrast, if the contact angle is higher than 90°, the emulsion will be w/o. In this case, it was intended to achieve an o/w emulsion because it is more pleasant cosmetologically speaking.

By the present analysis (Table 3), QSB particles have high wettability in all the oils but especially in CT, since the contact angles are lower than 30º. The contact angle of the solid particles with water is higher than 90º, which would indicate that QSB particles will stabilize more effectively w/o emulsions. Concerning the wettability results, it was concluded that CT was better suited for the oil phase.

Cork is a very light raw material; weighing just 0.16 g/cm^2^, it is practically impermeable to liquids and gases, thanks to suberin and ceroids, and it is highly resistant to moisture. Thus, this characteristic supports cork as a promising material to stabilize w/o Pickering emulsions.

However, there is a phenomenon known as phase inversion by which the dispersed phase becomes the continuous phase and vice versa, it can be considered a useful route to produce emulsion made of very fine droplets and it is widely used in fabrication of cosmetic products. Phase inversion can be brought about by changing the temperature of the system, by changing the volume fraction of the phases, by adding salts or by imposing particular flows [28]. In this case, the percentage of water we used for the formulations, relative to the quantity of oil phase, is much larger, which allows the emulsion to be o/w, reverting from the expected w/o.

### 3.3. Characterization of Emulsions Stabilized by QSB Particles

Macroscopic observation of the emulsion, after one week, revealed that emulsion was practically homogeneous and exhibited no separation of phases. The obtained emulsion was o/w type, confirming the theory described in the previous point. Based on the macro and microscopic observation and on the droplet size distribution, the oil phase of the emulsion stabilized by QSB particles was selected. CT was the best choice since it presented the smaller droplet size allied to a very good stability, based on the macroscopic observation of 2-week and 4-week emulsion, where it presented a homogenous aspect and no separation of phases (Table 4). In addition, in the microscopic observation of emulsion, the droplets were clearly visible, small and uniformly shaped.

### 3.4. Optimization Studies

#### Risk Analysis of CQAs

**Figure 2:**
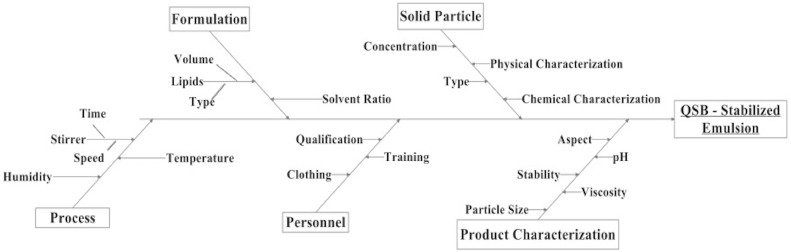
Ishikawa diagram illustrating factors that may have impact on the droplet size of a QSB-stabilized emulsion [29].

Factors potentially affecting the quality attributes of the Pickering emulsions were formulation and process related. Critical variables were identified and the effects of these variables on the droplet size were studied (Figure 2). This screening experimental design has the advantage of minimizing the number of experiments required to identify the most critical factors affecting the response. After the risk analysis and based on the literature and previous work, plus knowledge acquired in the laboratory during the experiment, we arrived to the conclusion that the major CQAs were the percentage of QSB particles and the volume of internal phase (percentage of oil phase) and those were the variables that were studied. The process variables have an impact on product quality; however, they were optimized previously, using the same conditions as described elsewhere [5].

The amount of QSB particles used in the designed experiments was estimated based on previous work [5], in which 5% amount of solid particles was selected as the CCD “0” level and 2.5 and 7.5 % as the “-α” and “α” levels, respectively.

### 3.5. Establishment of Design Space

#### 3.5.1. Response Surface Analysis

The data obtained by the experimental design was analyzed resorting to the MODDE® software and polynomial models were obtained. An ANOVA was also performed and the *p*-value was calculated for each variable. When the *p*-value found was equal or greater than 0.05, it was assumed that no significant effect was present. Moreover, the information derived from the models was expanded graphically by using isoresponsive curves. A good correlation was obtained between the observed and predicted values as indicated by the *R*^2^ value between 0.93 and 0.99 for all variables in the optimization study.

In Table 5, the coefficient column reflects each factor’s relative strength, the higher the absolute, the greater the effect of that factor on the response.

**Figure 3:**
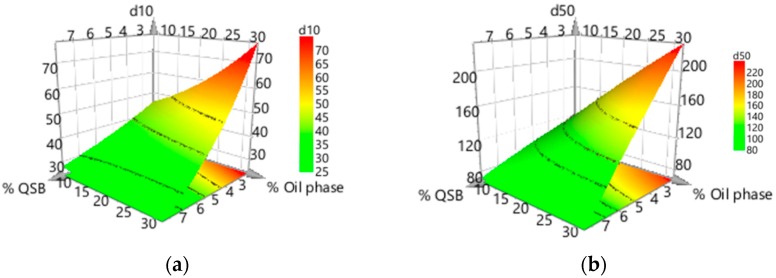

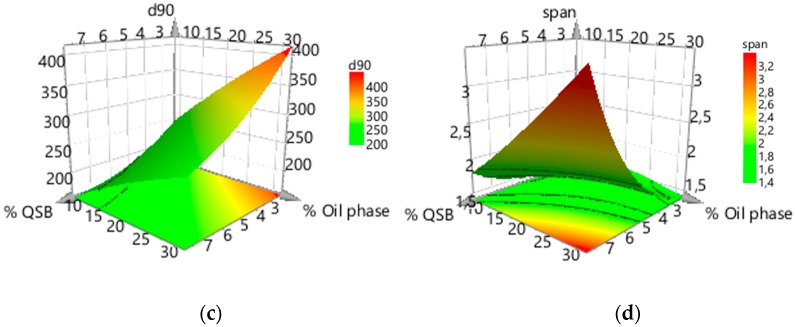
Isoresponse curves (graph floor) and response surface plots of relative size distribution (µm), respectively: (**a**) d(10), (**b**) d(50), (**c**) d(90) and (**d**) span, for formula optimization. % QSB—% Quercus Suber Bark particles.

A positive value indicates an effect that increases the response and a negative value represents an inverse relationship between the response and the factor. A general interpretation indicates that although an increase in QSB concentration and a decrease in the oil phase contributed to a narrower droplet size distribution, the former had a more dominant effect. In what concerns the formula optimization, the response surface plots are shown in Figure 3. 

Considering our formula optimization, the statistically significant variables (*p* < 0.05) that affected the droplet size distribution were concentration of QSB particles and volume of oil phase. Indeed, a negative correlation was observed for the percentage of QSB particles in the dependent variables d(10), d(50) and d(90), meaning that an increase in the QSB particles amount in the emulsion will result in a decrease in droplet size. According to several authors, the size of the droplets decreases with increasing solid particle concentration, leading to the formation of emulsions throughout the entire mixture volume without any coalescence or phase separation. However, if the droplet size approaches the size of the solid particles, flow oscillations are induced, leading to droplet break-up and increased polydispersity [30].

The percentage of oil phase also influenced the droplet size distribution, but with a positive correlation, which means that, with an increase in percentage of internal phase, the smaller droplets tend to aggregate, originating larger droplets, that will lead to higher values of droplet size. A positive correlation was found for Span, respecting both concentration of QSB particles and % of internal phase, meaning that an increase in QSB or oil phase concentration can lead to a wider droplet size distribution.

#### 3.5.2. Design Space

**Figure 4:**
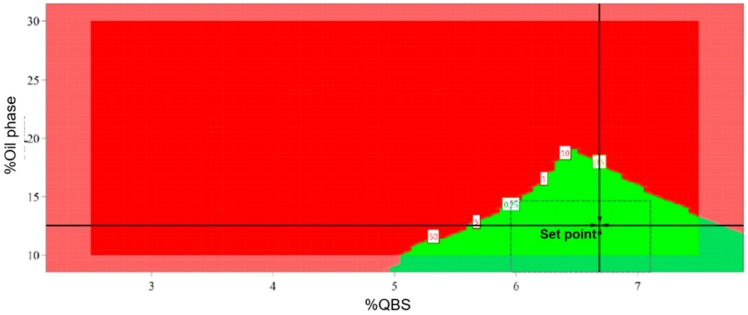
Plot evidence the design space for the QSB-based emulsion.

The design space (DS) concept is defined as the multidimensional combination and interaction of input variables and process parameters that have been demonstrated to provide assurance of quality [31]. In this study, response surface methodology was applied to establish the DS. The process key parameters that had been demonstrated to affect emulsions quality were used to construct the DS (Figure 4). 

Every single point corresponds to a combination of our studied variables. The green area corresponds to a range of combinations for which the droplet size remains within the pre-defined acceptable limits (Figure 3). This plot provides a range within the value of a critical parameter that will not affect the final response, meaning, as long as each variable is maintained within its range, the droplet size can be successfully predicted and controlled. The DS was established for formula optimization, which was delineated in the green region in Figure 3. The optimal conditions defined by the DS plot were 12.5% of CT and 6.7% of QSB, as shown in the set point of the previous figure.

### 3.6. Final Formulation

#### Physicochemical Characterization

The pH value was 5.42 ± 0.01, which is compatible with the pH of the human skin [32].

The viscosity profile of a formulation provides important information about the production, processing and performance. Structural differences in the formulations can be inferred from the flow curves. Rotational shear experiments measure the ability of a system to resist to structural deformation during the standardized shearing procedure. The flow curves showed that QSB-stabilized emulsion was a non-Newtonian fluid, which means that its viscosity is dependent on the shear rate. This emulsion was also characterized as shear thinning fluid, since a nonlinear curve was obtained and the apparent viscosity decreased with increased shear rate (shear thinning behaviour) [33,34].

**Figure 5:**
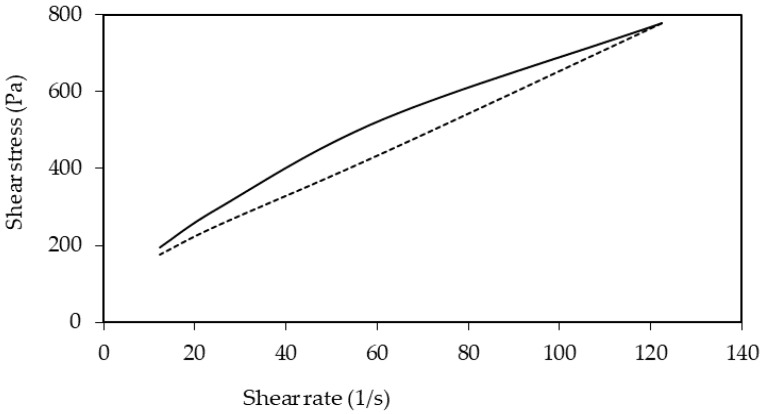
Flow curve of the QSB-based emulsion (line—up curve; dot—down curve).

In addition, the relation between the shear stress and the shear rate was probably also time-dependent, showing a thixotropic behaviour. Considering the time during which this formulation was submitted to different forces, it was possible to verify that the apparent viscosity was not only dependent on the shear rate magnitude, but also on the time of the shear rate application. In this context, the emulsion exhibited thixotropic behaviour because, observing the down curve of Figure 5, there is a slow recovery of the microstructure of the fluid to its initial state, taking a measurable time for the viscosity to recover [33,35]. As the emulsion behaved as a shear-thinning fluid, it is suitable for topical administration.

### 3.7. Stability Assessment of Optimized Formulation

After preparing the optimized formulation of Pickering emulsion stabilized by QSB particles (QSB-stabilized emulsion), given to us by the DS, it was important to assess its stability to see if the QSB-stabilized emulsion would maintain a cohesive structure and remain a stable emulsion. For this purpose, we prepared three batches of QSB-stabilized emulsion and measured their droplet size throughout a month. A plot was elaborated to compare the droplet size distribution between the three batches and, in the same batch, after 14 and 30 days. 

As expected, the three batches showed very similar profiles, which means they are reproducible, since they are all from QSB-stabilized emulsion. The curves also present very similar profiles of droplet size distribution, which means that, over time, the emulsion remained stable, with a cohesive structure. The more homogeneous the graph looks, the more stable the emulsion is. The droplet size distribution of QSB-stabilized emulsion can be defined as a Normal-Gaussian distribution. Moreover, a mono-modal distribution was observed, meaning that the emulsions remained stable for at least a month [36]. 

### 3.8. In Vitro Studies

#### 3.8.1. Cytotoxicity Assay

To predict the potential cytotoxicity of the QSB-stabilized emulsion, cell viability was evaluated using HaCaT cell line using an MTT assay. The cell viability of QSB-stabilized emulsion was 100.3 ± 6.6%. According to the OECD guideline, an irritant substance is predicted if the mean relative tissue viability is found below 50% of the mean viability of the negative controls for a 15–60 min of exposition time [37]. Thus, the QSB-stabilized emulsion can be intended as non-irritant and the amounts of QSB particles used can be considered safe. 

#### 3.8.2. Anti-Oxidant and Anti-Elastase Activity

**Figure 6:**
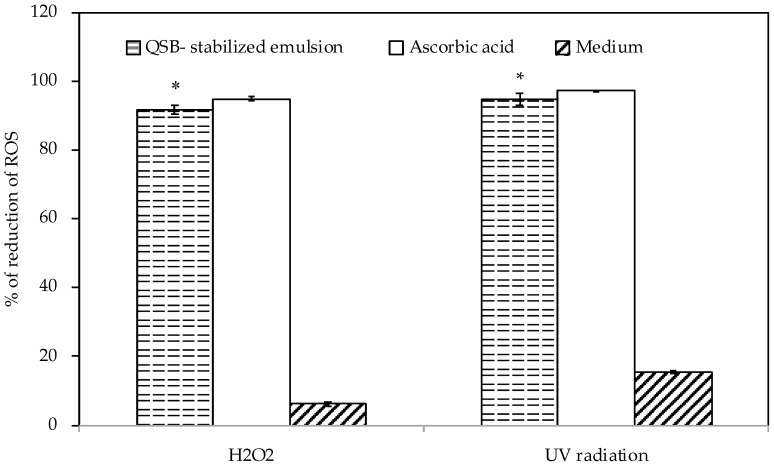
ROS (Reactive Oxygen Species) reduction after exposure to H_2_O_2_ of HaCaT cells and UV-B radiation, containing QSB-stabilized emulsion and ascorbic acid. The data are expressed as the mean of at least 12 replicate experiments ± standard deviation. Significance: (*) *p* < 0.05 versus positive control cells (ascorbic acid).

The possible protective effect of QSB-stabilized emulsion against oxidative damage in HaCaT cell lines was investigated in vitro. The QSB-stabilized emulsion has shown anti-oxidant activity (Figure 6) and the capacity to inhibit the oxidation of other molecules. This is supported by the knowledge of the existence of Quercus Suber Bark’s antioxidant proprieties [17]. 

Ascorbic acid was used as a positive control due to its potent antioxidant properties, measured by the capacity to reduce ROS species, and because it possesses a variety of other topical advantages including photoprotection from UVR, and improvement of a variety of inflammatory skin disorders [38].

As the statistical analysis indicates, the QSB-stabilized emulsion led to an evident reduction of ROS, although, when compared, not as accentuated as with ascorbic acid and with a higher standard deviation. This antioxidant phenomenon can probably be explained by the presence of certain composites of QSB. The presence of several phenolic acids in QSB was identified and, in several studies, these phenolic compounds revealed a wide variety of relevant properties, namely, their antioxidant and radical scavenger properties [17,39]. Suberin, a natural aliphatic-aromatic cross-linked polyester, is the main composite of QSB and was likewise shown to have antioxidant proprieties [39,40]. Another group of constituents of QSB identified to have antioxidant behavior was terpenes, one of the most extensive and varied structural compounds occurring in nature, providing relevant protection under oxidative stress [41].

The QSB-stabilized emulsion was evaluated for inhibitory activity on human neutrophil elastase and its IC50 was found to be 0.25 mg/mL. This anti-elastase phenomenon can probably be explained by the presence of certain phenolic compounds of QSB. Several studies showed that extracts containing gallic, protocatechuic and caffeic acids inhibit elastase activity. In addition, terpenoid compounds are elastase inhibitors, preventing the degradation of elastin fibrous structure in the dermal matrix [15]. 

### 3.9. In Vivo Compatibility and Sensorial Studies

In vivo safety testing can be used to generate product claims. HRIPT was conducted to justify the claim “dermatological tested”. During the HRIPT study, no reactions or skin sensitization/irritation were observed in the initial three weeks of contact or even after the final challenge contact. Thus, very good skin compatibility was obtained for QSB-stabilized emulsion. The cosmetic properties of the QSB-stabilized emulsion were assessed using a simple sensory questionnaire in which the basic characteristics, like application and skin feel, were evaluated by volunteers, during and after application. Results showed that QSB-FF met consumer appeal and acceptance requirements. The general opinion was truly positive and so QSB-FF presented the highest scores for spreadability and skin feel on application. It presented a low score for oiliness and stickiness. The fragrance was very much appreciated by the volunteers and they considered that it was easy to apply and quick to spread. In the after feel, volunteers reported hydration and softness and the intent to buy was somewhat high.

## 4. Conclusions

Accordingly, a novel Quercus suber-based Pickering emulsion for topical application was design and characterized. The QSB-stabilized emulsion developed met the predefined goal of using biocompatible organic particles to create a high skin compatible emulsion. As previously outlined, QSB-stabilized emulsion had the necessary characteristics to make it a suitable vehicle for topical delivery system, presenting compatible pH for the human skin and shear-thinning behaviour. The in vitro study revealed that QSB-stabilized emulsion shows anti-oxidant and anti-elastase activity. This can be explained by the presence of several phenolic acids, suberin and terpenes in Quercus Suber Bark. The in vivo compatibility study confirmed that QSB-stabilized emulsion is not skin irritant, being safe for human use. In addition, the sensorial analysis confirmed that QSB-stabilized emulsion was very appreciated by the volunteers. They considered it easy to apply and quick to spread, giving a feel of softness and hydration to the skin.

Our findings combined with data reported so far enrich existing knowledge about the potent anti-oxidant action of QSB and highlight that topical Pickering emulsion stabilized by eco-friendly particles could be a very promising area in the pharmaceutical and cosmetic industry.

## Figures and Tables

**Table 1:** QTPP of QSB-stabilized emulsions.

QTPP Element	Target	Reference
Route of administration	Topical	[21]
Dosage form	Emulsion (Pickering emulsion)	[5]
	Droplet size distribution:	[5]
	d(10)—30–50 µm d(50)—100–150 µm	
	d(90)—130–190 µm	
	Span—1.5–1.9	
Stability	Without separation of phases	[5]
In vitro studies	Efficacy: Anti-oxidant and anti-elastase activity Safety: Cytotoxicity assay	[15]
In vivo studies	Skin biocompatibility: Human repeat insult patch test (HRIPT)	[22]

**Table 2:** Design of experiments (QSB-stabilized emulsion optimization).

Exp Name	Run Order	Design Matrix		Experimental Matrix for Formula Optimization		
		QSB	Oil phase	%QSB	%Oil phase	% Aq. Phase
N1	1	−1	−1	2.50	10.00	87.50
N2	6	1	−1	7.50	10.00	82.50
N3	11	−1	1	2.50	30.00	67.50
N4	9	1	1	7.50	30.00	62.50
N5	7	−1.14744	0	2.13	20.00	77.87
N6	10	1.14744	0	7.87	20.00	72.13
N7	2	0	−1.14744	5.00	8.53	86.47
N8	8	0	1.14744	5.00	31.47	63.53
N9	3	0	0	5.00	20.00	75.00
N10	4	0	0	5.00	20.00	75.00
N11	5	0	0	5.00	20.00	75.00

**Table 3:** Contact angle of water, Caprylic/Capric Triglyceride (CT), Phenoxyethyl Caprylate (XC), Dimethicone and Cetyl Dimethicone with QSB (Quercus Suber Bark) particles (mean ± SD, *n* = 3).

Particles	Contact Angle (θ °)				
	Water	CT	XC	Dimethicone	Cetyl Dimethicone
QSB	97.3 ± 0.3	0.0 ± 0.0	8.0 ± 2.4	25.2 ± 8.7	10.4 ± 11.8

**Table 4:** Droplet size distribution of QSB-based emulsion.

Batch	Droplet Size Distribution (μm)			
	Span	d10	d50	d90
**T 0 days**				
1	1.6 ± 0.0	33.1 ± 0.2	88.4 ± 1.1	179.2 ± 2.7
2	1.7 ± 0.0	32.0 ± 0.3	87.3 ± 1.0	179.5 ± 2.8
3	1.6 ± 0.0	34.6 ± 0.2	90.7 ± 0.6	182.1 ± 1.3
**T 14 days**				
1	1.7 ± 0.0	30.1 ± 0.1	79.5 ± 0.5	161.2 ± 1.1
2	1.6 ± 0.0	32.3 ± 0.5	82.8 ± 3.2	161.6 ± 6.5
3	1.6 ± 0.0	29.5 ± 0.1	76.7 ± 0.4	153.0 ± 0.7
**T 30 days**				
1	1.6 ± 0.0	31.2 ± 0.1	82.90.4	166.3 ± 1.2
2	1.6 ± 0.0	34.5 ± 0.2	91.1 ± 0.6	180.6 ± 1.2
3	1.7 ± 0.0	30.1 ± 0.1	80.9 ± 0.4	164.4 ± 1.3

**Table 5:** Summary of regression analysis results for measured responses (central composite design), for formula optimization.

Formula Parameters	d(10)		d(50)		d(90)		Span	
	Coeff	± SE	Coeff	± SE	Coeff	± SE	Coeff	± SE
K	1.58	0.03	126.27	3.74	255.02	7.61	0.23	0.01
QSB	−0.16	0.02	−46.02	2.65	−52.63	5.39	0.09	0.01
Oil	NS	NS	23.81	2.65	60.74	5.39	0.05	0.01
Oil*Oil	NS	NS	NS	NS	NS	NS	NS	NS
QSB*QSB	NS	NS	NS	NS	NS	NS	0.06	0.01
Oil*QSB	NS	NS	−22.46	3.41	NS	NS	0.07	0.01

Oil—% Oil Phase; QSB—% Quercus Suber Bark particles. Coeff—Coefficient Scaled and centered; SE—Standard Error; k—Constant; NS (no significant)—*p* > 0.05.

## References

[1] Marto J., Ascenso A., Simões S., Almeida A.J., Ribeiro H.M. (2016). Pickering emulsions: Challenges and opportunities in topical delivery. Expert Opin. Drug Deliv..

[2] Ribeiro H.M., Morais J.A., Eccleston G.M. (2004). Structure and rheology of semisolid o/w creams containing cetyl alcohol/non-ionic surfactant mixed emulsifier and different polymers. Int. J. Cosmet. Sci..

[3] Bouyer E., Mekhloufi G., Rosilio V., Grossiord J.L., Agnely F. (2012). Proteins, polysaccharides, and their complexes used as stabilizers for emulsions: Alternatives to synthetic surfactants in the pharmaceutical field?. Int. J. Pharm..

[4] Marto J., Baltazar D., Duarte A., Fernandes A., Gouveia L., Militão M., Salgado A., Simões S., Oliveira E., Ribeiro H.M. (2015). Topical gels of etofenamate: In vitro and in vivo evaluation. Pharm. Dev. Technol..

[5] Marto J., Gouveia L.F., Jorge I.M., Duarte A., Gonçalves L.M., Silva S.M.C., Antunes F.E., Pais A.A.C.C., Oliveira E., Almeida A.J. (2015). Starch-based Pickering emulsions for topical drug delivery: A QbD approach. Colloid Surf. B: Biointerfaces.

[6] Marto J., Gouveia L.F., Gonçalves L., Chiari-Andréo B.G., Isaac V., Pinto P., Oliveira E., Almeida A.J., Ribeiro H.M. (2016). Design of novel starch-based Pickering emulsions as platforms for skin photoprotection. J. Photochem. Photobiol. B: Biol..

[7] Chevalier Y., Bolzinger M.A. (2013). Emulsions stabilized with solid nanoparticles: Pickering emulsions. Colloid. Surf. A: Physicochem. Eng. Asp..

[8] Matos M., Timgren A., Sjöö M., Dejmek P., Rayner M. (2013). Preparation and encapsulation properties of double Pickering emulsions stabilized by quinoa starch granules. Colloid. Surf. A: Physicochem. Eng. Asp..

[9] Binks B.P. (2002). Particles as surfactants—Similarities and differences. Curr. Opin. Colloid Interface Sci..

[10] Kaewsaneha C., Tangboriboonrat P., Polpanich D., Eissa M., Elaissari A. (2013). Preparation of Janus colloidal particles via Pickering emulsion: An overview. Colloid. Surf. A: Physicochem. Eng. Asp..

[11] Timgren A., Rayner M., Sjöö M., Dejmek P. (2011). Starch particles for food based Pickering emulsions. Procedia Food Sci..

[12] Marku D., Wahlgren M., Rayner M., Timgren A. (2012). Characterization of starch Pickering emulsions for potential applications in topical formulations. Int. J. Pharm..

[13] Rayner M., Marku D., Eriksson M., Dejmek P., Wahlgren M. (2014). Biomass-based particles for the formulation of Pickering type emulsions in food and topical applications. Colloid. Surf. A: Physicochem. Eng. Asp..

[14] Laredj-Bourezg F., Chevalier Y., Boyron O., Bolzinger M.A. (2012). Emulsions stabilized with organic solid particles. Colloid. Surf. A: Physicochem. Eng. Asp..

[15] Carriço C., Ribeiro H.M., Marto J. (2018). Converting cork by-products to ecofriendly cork bioactive ingredients: Novel pharmaceutical and cosmetics applications. Ind. Crop Prod..

[16] Jové P., Olivella A.C., Cano L. (2011). Study of the variability in chemical composition of bark layers of Quercus Suber L. from different production areas. BioResources.

[17] Santos S., Pinto. P., Silvestre A., Neto C.P. (2010). Chemical composition and antioxidant activity of phenolic extracts of cork from *Quercus suber* L.. Ind. Crop Prod..

[18] Castola V., Marongiu B., Bighelli A., Floris C., Laï A., Casanova J. (2005). Extractives of cork (*Quercus suber* L.): Chemical composition of dichloromethane and supercritical CO_2_ extracts. Ind. Crop Prod..

[19] Subhashini S., Begum S.F.M., Rajesh G. (2016). Antimicrobial characterisation combining spectrophotometric analysis of different oak species. Int. J. Herb. Med..

[20] Khennouf S., Benabdallah H., Gharzouli K., Amira S., Ito H., Kim T.H., Yoshida T., Gharzouli A. (2003). Effect of Tannins from Quercus suber and Quercus coccifera Leaves on Ethanol–Induced Gastric Lesions in Mice. Int. J. Agric. Food Chem..

[21] Coquet C., Bauza E., Oberto G., Berghi A., Farnet A.M., Ferré E., Peyronel D., Dal Farra C., Domloge N. (2005). Quercus suber cork extract displays a tensor and smoothing effect on human skin: An in vivo study. Drugs Exp. Clin. Res..

[22] Marto J., Gonçalves L.M., Fitas M., Pinto P., Almeida A.J., Ribeiro H.M. (2018). Safety assessment of starch–based personal care products: Nanocapsules and pickering emulsions. Toxicol. Appl. Pharm..

[23] Marto J., Ascenso A., Gonçalves L.M., Gouveia L.F., Manteigas P., Pinto P., Oliveira E., Almeida A.J., Ribeiro H.M. (2016). Melatonin-based Pickering emulsion for skin’s photoprotection. Drug Deliv..

[24] Marzulli F.N., Maibach H.I. (1976). Contact allergy: Predictive testing in man. Contact Dermat..

[25] EC, EC (2009). Regulation (EC) No 1223/2009 of the European Parliament and of the Council of 30 November 2009 on Cosmetic Products.

[26] Molina J.M., Narciso J., Weber L., Mortensen A., Louis E. (2008). Thermal conductivity of Al-SiC composites with monomodal and bimodal particle size distribution. Adv. Mater. Res.-Switz..

[27] Ruckenstein E. (1996). Microemulsions, Macroemulsions, and the Bancroft Rule. Langmuir.

[28] Preziosi V., Perazzo A., Caserta S., Tomaiuolo G., Guido S. (2013). Phase Inversion Emulsification. Chem. Eng. Trans..

[B29-pharmaceutics-11-00131] 29.International Conference on Harmonisation (ICH) of Technical Requirement for Registration of Pharmaceuticals for Human Use, Pharmaceutical Development, Q8 (R2). 2009.

[30] Lam S., Velikov K.P., Velev O.D. (2014). Pickering stabilization of foams and emulsions with particles of biological origin. Curr. Opin. Colloid Interface Sci..

[31] Lionberger R.A., Lee S.L., Lee L., Raw A., Lawrence X.Y. (2008). Quality by Design: Concepts for ANDAs. Int. J. AAPS.

[32] Schmid-Wendtner M.H., Korting H.C. (2006). The pH of the Skin Surface and Its Impact on the Barrier Function. Skin Pharmacol. Physiol..

[33] Marcottea M., Hoshahilia A.R.T., Ramaswamy H.S. (2001). Rheological properties of selected hydrocolloids as a function of concentration and temperature. Food Res. Int..

[34] Zhong L., Oostrom M., Truex M.J., Vermeul V.R., Szecsody J.E. (2013). Rheological behavior of xanthan gum solution related to shear thinning fluid delivery for subsurface remediation. Int. J. Hazard. Mater..

[35] Mendes P.R.S. (2009). Modeling the thixotropic behavior of structured fluids. J. Non-Newton Fluid Mech..

[36] Rykaczewski K., Paxson A.T., Anand S., Chen X., Wang Z., Varanasi K.K. (2013). Multimode Multidrop Serial Coalescence Effects during Condensation on Hierarchical Superhydrophobic Surfaces. Langmuir.

[37] OECD (2010). Test No. 439: In Vitro Skin Irritation Reconstructed Human Epidermis Test Method: Reconstructed Human Epidermis Test Method.

[38] Murray J.C., Burch J.A., Streilein R.D., Iannacchione M.A., Hall R.P., Pinnell S.R. (2008). A topical antioxidant solution containing vitamins C and E stabilized by ferulic acid provides protection for human skin against damage caused by ultraviolet irradiation. J. Am. Acad. Dermatol..

[39] Lee J.Y., Yoon J.W., Kim C.T., Lim S.T. (2004). Antioxidant activity of phenylpropanoid esters isolated and identified from Platycodon grandiflorum A. DC. Phytochemistry.

[40] Dönmez I.E., Hemming J., Willför S. (2016). Bark extractives and suberin monomers from Arbutus andrachne and Platanus orientalis. BioResources.

[41] Gonzalez-Burgos E., Gomez-Serranillos M.P. (2012). Terpene Compounds in Nature: A Review of Their Potential Antioxidant Activity. Curr. Med. Chem..

